# Ischemia–Reperfusion Injury in Kidney Transplantation: Mechanisms and Potential Therapeutic Targets

**DOI:** 10.3390/ijms25084332

**Published:** 2024-04-14

**Authors:** Francesco Lasorsa, Monica Rutigliano, Martina Milella, Antonio d’Amati, Felice Crocetto, Savio Domenico Pandolfo, Biagio Barone, Matteo Ferro, Marco Spilotros, Michele Battaglia, Pasquale Ditonno, Giuseppe Lucarelli

**Affiliations:** 1 Department of Precision and Regenerative Medicine and Ionian Area-Urology, Andrology and Kidney Transplantation Unit, University of Bari “Aldo Moro”, 70124 Bari, Italy; 2 Department of Precision and Regenerative Medicine and Ionian Area-Pathology Unit, University of Bari “Aldo Moro”, 70124 Bari, Italy; 3 Department of Neurosciences, Science of Reproduction and Odontostomatology, University of Naples Federico II, 80131 Naples, Italy; 4 Department of Urology, University of L’Aquila, 67010 L’Aquila, Italy; 5 Division of Urology, Department of Surgical Sciences, AORN Sant’Anna e San Sebastiano, 81100 Caserta, Italy; 6 Division of Urology, European Institute of Oncology, IRCCS, 71013 Milan, Italy

**Keywords:** kidney transplantation, graft, ischemia, reperfusion, injury, IRI, complement

## Abstract

Kidney transplantation offers a longer life expectancy and a better quality of life than dialysis to patients with end-stage kidney disease. Ischemia–reperfusion injury (IRI) is thought to be a cornerstone in delayed or reduced graft function and increases the risk of rejection by triggering the immunogenicity of the organ. IRI is an unavoidable event that happens when the blood supply is temporarily reduced and then restored to an organ. IRI is the result of several biological pathways, such as transcriptional reprogramming, apoptosis and necrosis, innate and adaptive immune responses, and endothelial dysfunction. Tubular cells mostly depend on fatty acid (FA) β-oxidation for energy production since more ATP molecules are yielded per substrate molecule than glucose oxidation. Upon ischemia–reperfusion damage, the innate and adaptive immune system activates to achieve tissue clearance and repair. Several cells, cytokines, enzymes, receptors, and ligands are known to take part in these events. The complement cascade might start even before organ procurement in deceased donors. However, additional experimental and clinical data are required to better understand the pathogenic events that take place during this complex process.

## 1. Introduction

Kidney transplantation (KT) offers a longer life expectancy and a better quality of life than dialysis to patients with end-stage kidney disease (ESKD). In 2022, 2033 kidney transplantations were performed in Italy, 336 of whom were living-donor KTs. To face the large number of candidates on the waiting lists, most countries considered enlarging the pool of donors by accepting extended criteria donors (ECD) and donors after circulatory death (DCD) [1]. ECD is referred to as donors > 60 years or >50 years with two additional features (serum creatinine > 1.5 mg/dL, cerebrovascular accident as death cause, history of hypertension). Many studies highlighted that ECD carried a higher risk of delayed graft function (DGF), primary nonfunction (PNF), and acute rejection with chronic graft failure [2]. Delayed graft function is attributed to grafts that regain function after a few days to weeks, whereas those organs that never function are indicated as primary nonfunction (PNF). Graft nephrectomy is usually indicated for PNF [3]. The risk of DGF is reported to be two-fold higher in organs from DCD than from donation after brain death (DBD) [4,5]. Ischemia–reperfusion injury (IRI) is thought to be a cornerstone in delayed or reduced graft function and increases the risk of rejection by triggering the immunogenicity of the organ [6]. The initial acute tubular necrosis (ATN) is followed by the repair of acutely injured structures and progressive functional stabilization of the organ. During this period, ATN can be exacerbated by nephrotoxic drugs such as calcineurin inhibitors. Severe IRI and immune response can even cause irreversible nephron loss (glomerular sclerosis and proximal tubular injury) that eventually triggers chronic allograft dysfunction. It has been largely described as the poorer long-term graft survival in recipients who experienced DGF or acute rejection.

## 2. Pathophysiology of IRI

Ischemia–reperfusion injury (IRI) is an unavoidable event that happens when the blood supply is temporarily reduced and then restored to an organ. Depending on the clinical setting, kidneys are vulnerable to cold or warm ischemia. Cold ischemia happens during transplantation following organ procurement and during its static preservation, while warm ischemia happens during vascular anastomosis. IRI is the result of several biological pathways, such as transcriptional reprogramming, apoptosis and necrosis, innate and adaptive immune responses, and endothelial dysfunction. Reduced blood supply alters cell metabolism because of reduced oxygen supply and removal of end-products. The switch toward anaerobic cell metabolism leads to lower adenosine triphosphate (ATP) production, lactate accumulation, and intracellular acidosis. Morphological rearrangement occurs because of limited Na^+^/K^+^, Na^+^/H^+^, and Ca^2+^ATPase activity and cytoskeletal protein disruption. Sodium and calcium ions accumulate: Ca^2+^-dependent proteases (as calpains) and lysosomal enzymes activate. Intracellular acidosis limits calpains activity, which is fully restored during reperfusion. Restored oxygen supply (normoxemia) is responsible for the harmful production of reactive oxygen species (ROS). Indeed, it has been supported that ROS production is higher during reperfusion because of reduced cytochromes and xanthine oxidase and the activation of nicotinamide adenine dinucleotide phosphate (NADPH) oxidase. ROS can alter membranes and DNA through lipid peroxidation and protein carbonylation. Calcium and ROS overload in association with mitochondrial dysfunction cause the opening of mitochondrial permeability transition pores (mPTPs) and the subsequent release of cytochrome C, mitochondrial DNA (mtDNA), and succinate. Necrosis, apoptosis (both intrinsic and extrinsic pathway), and necroptosis events initiate [7]. The extrinsic pathway seems to predominate during the reperfusion phase. In this scenario, the peritubular capillaries in the kidney have a restricted capacity to repair after damage. During ischemia, the conformational changes of endothelial cells and their loss of cytoskeleton and glycocalyx increase their permeability, so fluids accumulate in the interstitium. Vasoconstriction is further supported by lower nitric oxide levels (nitric oxide synthase is downregulated) whereas endothelin-1 (ET1) and platelet-derived growth factor (PDGF) are synthetized from endothelial cells. The latter are more vulnerable to the vasoconstrictive action of thromboxane-A2, prostaglandins, and angiotensin-II. Endothelial-to-mesenchymal transition (EndMT) can support interstitial fibrosis and tubular atrophy (IFTA) after capillary damage. Like inflammation, IRI in the kidney causes P-selectin to be expressed on endothelial cells. This glycoprotein causes leukocyte chemotaxis and immune cell transmigration to the interstitium by interacting with P-selectin glycoprotein 1 (PSGL1), which is expressed on the surface of leukocytes. Leukocytes can adhere to endothelial cells through the β2-integrins and intercellular adhesion molecule 1 (ICAM1) interface, and they can transmigrate into the interstitial compartment through the platelet endothelial cell adhesion molecule (PECAM1) relationship.

**Figure 1:**
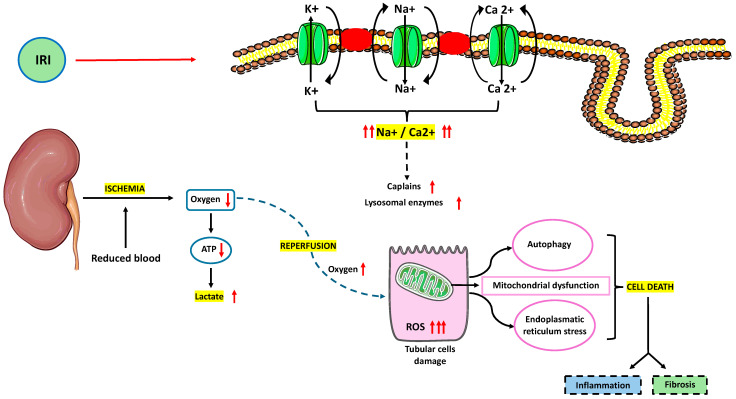
Pathophysiology of ischemia–reperfusion injury (IRI).

Ischemia and reperfusion also affect the genomic level since ROS may affect the expression of mRNA and non-coding RNAs. Many of the up-regulated genes are involved in cell survival, cell surface signaling, response to oxidative stress, and inflammatory response. In turn, genes involved in ion transport are typically downregulated [8]. Park et al. performed RNA sequencing (RNA-seq) to examine gene expression variations separately during ischemia and during the reperfusion phase, compared to normal tissue. This study showed that during ischemia, metabolism-related pathways were enriched, such as melatonin/serotonin degradation (scavengers of free radicals), lipid metabolism (FXR/RXR pathway), bupropion/acetone degradation, and estrogen biosynthesis. Ischemia also enhanced pathways related to apoptosis, fibrosis, and adipogenesis. In the reperfusion phase, immune-response pathways (inflammasome and antigen presentation) were dysregulated. Additionally, processes related to cell development, growth, and migration were affected [9]. Recently, Zhang et al. investigated the role of endoplasmic reticulum stress (ERS) during renal IRI. The endoplasmic reticulum (ER) plays a crucial role in protein synthesis, folding, and definitive maturation. Unfolded and misfolded proteins accumulate because of impaired ER following hypoxia, ischemia, oxidative stress, or metabolic abnormalities. The role of ERS during IRI appears discrepant, nevertheless. Transcriptomic analysis revealed that inducers and downstream targets of ERS were upregulated: PPP1R15A, JUN, and ATF3 above all. These three genes might interact with kidney injury-related pathways (apoptosis, inflammatory response, oxidative stress, and pyroptosis), and their inhibition alleviated IRI. Additionally, higher levels of these genes were associated with poor graft outcomes [10] (Figure 1).

## 3. Metabolic Reprogramming during IRI

A large amount of oxygen is constantly required for renal physiological functions. Different factors are supposed to make the kidneys vulnerable to hypoxia, such as low medullary blood flow to support urinary concentration, limited angiogenesis, and limited vasodilator response to hypoxic conditions. Under hypoxia, proximal tubular cells (PTC) secrete several inflammatory mediators such as IL-8, monocyte chemoattractant protein 1 (MCP-1), tumor necrosis factor α (TNFα), and regulated upon activation, normal T-cells expressed and secreted (RANTES) [8]. Tubular cells mostly depend on fatty acid (FA) β-oxidation for energy production since more ATP molecules are yielded per substrate molecule than glucose oxidation [11,12,13,14]. FA are oxidized into acetyl-coenzyme A (CoA), and most of the reactions occur in the mitochondria. Mitochondrial damage includes a lower number of organelles, their swelling, and disruption of cristae during IRI, and other forms of acute kidney injury (AKI). As mentioned above, disrupted mitochondria release ROS, cytochrome C, and mtDNA. Lipidomic analysis revealed the accumulation of FA, triglycerides, and ceramides, while acyl-CoAs and acyl-carnitines decreased. The suppression of β-oxidation is suggested by the downregulation of carnitine palmitoyltransferase 1A (CPT1A), long-chain acyl-CoA dehydrogenase (LCAD), and medium-chain acyl-CoA dehydrogenase (MCAD). Ma et al. showed a significant reduction in the activity of AMP-activated protein kinase (AMPK), a normal sensor of energy conditions. ATP depletion and AMP accumulation result in the activation of AMPK and its myriad of downstream effectors to enhance ATP-producing catabolic reactions and to abolish anabolism. The synthesis of ceramides has been associated with AMPK downregulation and the activation of protein phosphatase PP2A, that PP2A further dephosphorylates AMPK. Injured mitochondria are normally discharged via mitophagy/autophagy and are promptly replenished via biogenesis. Mitochondria and NADPH oxidase (NOX) are the main sources of ROS. Superoxide anion is regarded as the most dangerous mitochondrial ROS (mtROS). Mitophagy is the autophagic mechanism of removal of aged and damaged mitochondria [15,16,17]. A ubiquitin-dependent and a ubiquitin-independent pathway are classically described. PTEN-induced putative kinase 1 (PINK1) and Parkin control the first one. PINK1 is a serine/threonine kinase that is normally shuttled into the mitochondria and is cleaved by the intramembrane serine protease presenilin-associated rhomboid-like (PARL). Upon injury, the loss of membrane potential results in PINK1 accumulation at the level of the mitochondrial outer membrane (MOM). Then, Parkin (PRKN- a cytosolic ubiquitin E3 ligase) is recruited and targets several substrates as mitofusin 2 (Msn2), voltage-dependent anion-selective channel protein (VDAC) and dynamin-1-like protein (DRP1). Thus, mitophagy receptors (optineurin, p62, and NBR1) are activated by ubiquitinated proteins. Mitochondria can bind to autophagosomes through LC3 and are finally engulfed by lysosomes [18]. The ubiquitin-independent pathway depends on mitophagy receptors on MOM as BNIP3 (BCL2 interacting protein 3), BNIP3L/NIX (BNIP3-like), FUNDC1 (FUN14 domain containing 1), AMBRA1 (autophagy and beclin 1 regulator 1), or on the inner membrane as PHB2 (prohibitin 2). Through LC3, they directly link damaged organelles to autophagosomes [19,20,21]. Increased ROS production, apoptosis, and tubulointerstitial inflammation were noted in mice models of IRI with deficient BNIP3, PINK1, and/or PRKN [22,23]. Consequently, the PINK1-Parkin pathway’s upregulation of mitophagy increased cell survival, reduced ROS generation, and enhanced mitochondrial function [24]. However, other networks have been identified as cardiolipin-mediated and ubiquitin-mediated mitophagy. Cardiolipin (CL) is almost exclusively located at the inner membrane. Phospholipid scramblase 3 (PLSCR3) allows its translocation to the outer leaflet of the membrane to interact with LC3 under stress conditions [25]. Additional ubiquitin-ligases other than PRKN can take part in this process, such as MUL1 (mitochondrial E3 ubiquitin protein ligase 1) [26]. Mitophagy regulator proteins are also involved in mitochondrial fusion and fission. Through fusion, two previously independent structures combine to form a single mitochondrion. When the potential of the mitochondrial membrane is diminished, fission creates one or more daughter organelles and separates them so that autophagy can remove them. FUNDC1 seems to orchestrate fission/fusion with mitophagy. This receptor anchors optic atrophy 1 (OPA1), a dynamin-related GTPase, toward the inner surface of the MOM under normal conditions. Disassembly of the FUNDC1–OPA1 complex and recruitment of DRP1 facilitate mitochondrial fission and mitophagy in response to mitochondrial stress [27]. Guanine is most likely to be oxidized to 8-oxoguanine (8-oxoG) under stress conditions. 8-Oxoguanine DNA glycosylase (OGG1) removes 8-oxoG via base excision repair of DNA and has been found to be upregulated in renal IRI, albeit its role remains unclear. Human OGG1α isoform is located in the nucleus, cytoplasm, and mitochondria, whereas OGG1β is only in the mitochondria. Recently, Zhao et al. investigated the role of OGG1 during renal IRI. OGG1 expression rose continuously as reperfusion time increased, but there was no corresponding increase in its base repair activity. They also reported that OGG1 interferes with mitophagy by limiting PINK1 translocation to the mitochondria. Accordingly, OGG1 inhibition or knockdown alleviated renal IRI-enhancing mitophagy [28]. Evidence supports the interaction between mitophagy and other forms of cell death (apoptosis, pyroptosis, and ferroptosis) during acute kidney injury (AKI). Iron accumulation, lipid peroxidation, and plasma membrane rupture define ferroptosis. During IRI, the activation of ferroptosis is contributed by several pathways. Ferritin is an intracellular iron-storage protein, and its degradation (ferritinophagy) is indirectly triggered by ischemia/reperfusion [29]. As a result of the oxidative stress, lower glutathione (GSH) content blocks the activity of glutathione peroxidase 4 (GPX4) with subsequent accumulation of dangerous lipid peroxides and the induction of ferroptosis. Pannexin 1 is a membrane channel and can activate apoptosis and autophagy. In a rodent model of IRI, its silencing decreased lipid peroxidation and decreased tubular cell apoptosis [30]. On the other hand, the administration of recombinant human augmenter of liver regeneration (ALR) also improved renal function because of its anti-apoptotic and anti-oxidative properties on tubular cells [31]. PGC-1α is referred to as a master regulator of mitochondrial biogenesis. Even histologically normal renal areas show its suppressed expression during AKI. Moreover, AMPK silencing has been linked to lower levels of pSer555-unc-51, like autophagy activating kinase 1 (ULK1), which controls autophagy. The authors also recognized that AMPK enhances β-oxidation and improves autophagy/mitophagy, thus supporting tubular cell recovery from energy stress and survival. Considering that AMPK activators (i.e., AICAR and metformin) can exert a protective role during IRI, they encouraged their putative therapeutic approach [32]. Hypoxia-inducible factor (HIF) plays a key role during hypoxia. Under normoxic conditions, HIF-1α is rapidly degraded in the proteasome after ubiquitination. Prolyl hydroxylases (PHD) hydroxylate specific proline residues in the presence of 2-oxoglutarate (2OG), O_2_ molecules, and ferrous iron (Fe^2+^). PHD activity is required for E3-ubiquitin ligase von Hippel–Lindau (VHL). Therefore, under hypoxia, HIF-1α is stabilized, and it dimerizes with HIF-1β to form HIF-1. The latter translocates to the nucleus and modulates gene expression by binding to hypoxia response elements (HRE). The expression of HIF-2α is further induced under this environmental condition. HIF-1 facilitates the transition to anaerobic glucose metabolism (glycolysis) and may hinder mitochondrial biogenesis and enhance mitophagy, whereas HIF-2α controls the autophagic removal of damaged peroxisomes (pexophagy) [33].

## 4. Immune Response during IRI

Upon ischemia–reperfusion damage, the innate and adaptive immune system activates to achieve tissue clearance and repair. Several cells, cytokines, enzymes, receptors, and ligands are known to take part in these events. Toll-like receptors (TLRs) are expressed in the cytosol and on the plasmatic membrane of leukocytes, monocytes, dendritic cells, and endothelial cells. TLRs are considered the main pattern recognition receptors (PRRs) and can bind to a plethora of microbial products that are generally referred to as pathogen-associated molecular patterns (PAMPs). Besides PAMPs, TLR4 and TLR2 also recognize endogenous ligands named danger-associated molecular patterns (DAMPs): heat shock proteins, high mobility group box 1 (HMGB1), and breakdown products of fibronectin, hyaluronic acid, and heparan sulfate. mRNA for all TLRs can be detected during IRI, albeit TLR2 and TLR4 are constitutively expressed in proximal and distant tubules, in the thin limb of the loop of Henle, and in the collecting ducts [34]. Interestingly, tubular epithelial cells upregulate TLR4 and TLR2 during ischemia, which is responsible for the activation of nuclear factor kappa-light-chain-enhancer of activated B cells (NF-kB), IFN-regulatory factor 3 (IRF3), an inhibitor of nuclear factor-kB kinase (IKK) and TANK-binding kinase-1 (TBK1) [35]. Both TLR4 knockout mice and kidneys from donors with TLR4 loss of function exhibited lower levels of cytokines and better immediate graft function [36]. TLR4 activation results in the release of many proinflammatory mediators (IL-1β, IL-6, TNF-α, macrophage inflammatory protein 2- MIP2) and the expression of molecules involved in leukocytes rolling and migration toward renal interstitial space (E-selectin and vascular cell adhesion molecule 1-VCAM1). Neutrophils and macrophages are also activated by this transmembrane receptor: cytokines, proteolytic enzymes, and ROS are released and further foster kidney damage. So, in the case of TLR4 knockout, their expression was silenced, and interstitial infiltration of macrophages and neutrophils was reduced [37,38]. Upregulation of TLR2 also supports the burst of the inflammatory response during IRI [39]. Transgenic mice were also used to assess the role of TLR2 during IRI: TLR2 knockout were better protected. Rusai et al. compared the results of single TLR2 or TLR4 knockouts to double TLR2/4 ones. Surprisingly, no increased protection was noted when both TLR2 and TLR4 were deleted. TLR2 silencing was also associated with reduced graft fibrosis (collagen I and III); therefore, it may ameliorate kidney chronic allograft dysfunction [40,41].

The first immune cells that arrive at the site of damage are neutrophils, which may play a role in causing renal injury by secreting ROS and proteases and clogging renal microvessels. In addition, they can also release cytokines that draw in additional neutrophils to form a positive feedback loop, which exacerbates tissue damage. While uncommon in healthy kidneys, macrophages become predominant in postischemic kidneys. Then, in a mouse model of renal IRI, suppression of macrophage infiltration reduced kidney damage [42]. Once DCs are activated, they synthesize many soluble mediators such as TNF-α, IL-6, monocyte chemoattractant protein 1 (MCP-1), and chemokine ligand 5 (CCL5). DCs activation was highlighted to start already in the donor after brain death because of oxidative stress and complement cascade. Poorer graft outcome has been associated with a higher density of DCs [43]. Dendritic cells (DCs) play a pivotal role to induce the adaptive immune response by processing and presenting antigens to B and T lymphocytes. T-cell activation demands T-cell receptor (TCR) to bind major-histocompatibility complex (MHC) on APCs. During transplantation, T cells can either directly interact with allogenic MHC on the donor APCs or indirectly with donor antigens after being processed and exposed to recipient MHC. Nevertheless, CD4 and CD8 co-stimulation is necessary to fully activate T cells. CD4+ (Th) cells bind to class II MHC, whereas CD8+ (T cytotoxic) cells bind to class I MHC. Non-polymorphic domains of MHC are recognized by these coreceptors. CD8+ T lymphocytes eliminate their targets through the FAS–FASL axis, granzyme, or perforin-mediated apoptosis. The cells have the potential to exert opposite effects based on their polarization [44]. It is widely accepted that T cells participate in IRI since T-cell-deficient mice experience reduced kidney injury during ischemia–reperfusion [45]. Th1 cells release IFN-γ, support the activation of macrophages, and the production of lysosomal enzymes, NO, and ROS. Th1 cells appear prevalent during DGF. In Th17 cells, IL-17 and IL-22 are downstream effectors of signal transducer and activator of transcription 3 (STAT3) transcription factor and the retinoic acid-related orphan receptor t (RORγt). Evidence suggests that STAT3 deficiency provides protection against IRI, as STAT3-knockout mice have decreased Th17 activity and a reduced inflammatory response [46,47]. FoxP3 T cells (T regs) have a protective role during IRI owing to anti-inflammatory cytokines (IL-10 and TGF-β), which turn off immune responses and support tissue repair by activating macrophages and fibroblasts. Therefore, graft tolerance and reduced IRI might be induced by T regs [48,49].

Kidneys have been demonstrated to be enriched with activated B cells during IRI that play a role both in the innate and adaptive immune response. CXCL13 possibly orchestrates B cell recruitment. TLRs can recognize damage-associated molecular patterns (DAMPs) and let B cells release polyreactive IgM, cytokines, and chemokines (innate response) [50]. Indeed, B cell-deficient mice revealed lower levels of chemokine CCL7 that recruits neutrophils and monocytes to injured tissues. The transcriptomic analysis further confirmed higher expression of CCL7 in human kidneys during IRI than in physiological conditions [51]. B cell receptor (BCR) stimulation and co-stimulatory molecules are necessary for the activation of naïve B cells, which then participate in adaptive immunity through antibody production. T–B cell interaction exists since Th1 and Th2 can modulate B cell immunoglobulin class switch and their effects. In the later stages of IRI, B and T lymphocytes were the most abundant immune cells and were organized into highly vascularized ectopic lymphoid structures (tertiary lymphoid organs) in clusters of resolved tubular injury. Finally, tertiary lymphoid organs (TLO) in grafts can lead to the transition from AKI to chronic kidney disease (CKD).

## 5. Complement System and IRI

A complement system is referred to as a group of approximately 50 elements, including circulating factors, membrane-bound receptors, activators, and regulators. Classical (CP), alternative (AP), and lectin (LP) pathways have been described, albeit all of them lead to the activation of C3 and to the deposition of membrane attack complex (MAC) on the target. Despite being mostly produced in the liver, complement elements can be locally produced by a plethora of cells (including kidney tubular epithelial cells). Rodent models were used to investigate the role of locally produced complement fragments. Grafts from C3-deficient donors developed a weakened immune response against allograft antigens because of defective T cell priming. Likewise, MHC II is not upregulated by DCs from C3, Factor B, or C3aR knockout mice or in DCs exposed to a C3aR antagonist. This results in inadequate alloreactive CD4+ T cell response and deficient T cell priming. Overall, graft survival was prolonged [52]. Additionally, it has been discovered that the key complement proteins C3 and C5, along with their activation components, receptors, and regulators, are active intracellularly as the complosome to control cell metabolism and physiology. Intracellular C3 is cleaved by cathepsin L (CTSL) in resting T cells. Upon T cell activation, intrinsic C3a and C3b translocate to the surface and bind to C3aR and CD46. Intracellularly generated C3b has been unveiled to increase the expression of amino acid transporter LAT1 and glucose transporter 1 (GLUT1) and to trigger mTORC1 signaling for the Th1 response [53]. Moreover, the engagement of C5aR1 contributes to the production of IFN-γ, whereas C5aR2 negatively regulates it [54].

Lasorsa et al. recently provided an extensive review to explore the role of the complement system in kidney diseases, transplantation, and cancer [55]. The complement cascade might start even before organ procurement in deceased donors [56]. Pre-existing medical conditions, hemodynamic changes, and warm ischemia (in DCD donors) account for complement activation after the release of DAMPs and inflammatory responses. Renal biopsies from DBD donors revealed C3d deposition, as well as serum from deceased donors, revealed complement activation via an alternative pathway [57]. Compared to living donors, DBD donors produced higher serum levels of C5a and sC5b-9 and were associated with a higher risk of rejection [58]. High levels of sC5b-9 were measured only in recipients from deceased donors. Consistently, pre-treatment of DBD donors with a C1 inhibitor or with monoclonal antibodies against Factor B improved graft functions in animal models [59,60]. Remarkably, underlying nephropathies (atypical hemolytic uremic syndrome, C3 glomerulopathy, diabetes) and maintenance hemodialysis are responsible for the activation of the complement system in recipients even before an available transplantation [61].

During ischemia, metabolic reprogramming creates an acidic environment that interferes with complement regulation and activates AP. Ischemic tubular, endothelial, and perivascular cells massively release DAMPs (hyaluronic acid, fibronectin, and DNA) which foster complement cascade since they are detected by C1q, mannose-binding lectin (MBL), collectins, ficolins, and C3b. The leading pathways implicated in abnormal complement activation during renal IRI are AP and LP, while the role of CP is limited. C4 deficiency did not ameliorate IRI. As for the AP, Factor B (FB)-deficient mice were protected from kidney damage as wild-type (WT) mice after anti-FB treatment. Severe kidney injury was noted in mice deficient in Factor H (FH), a negative AP regulator [62]. However, detrimental effects have been reported in the case of the lack of properdin (an AP-positive regulator), which might have a protective role in limiting inflammation [63]. During IRI, tubular epithelial cells overexpress collectin-11 (CL11). CL11 is a soluble pattern recognition receptor (PRR) that can detect extracellular DNA and fucosylated molecules on hypoxic cells. Thereafter, CL11 can activate mannan-binding serin protease (MASP), thus the following cascade. One putative effective therapeutic strategy against IRI might be to block the terminal pathway of the complement. Complement regulators CD55 and CD59 are essential during the process. Genetic deficiency aggravated kidney injury, which was reduced by their overexpression or recombinant CD55 administration by preventing C3 and C9 deposition [64]. CD55 deficiency in APC or T cells leads to higher T cell proliferation because of uncontrolled C3 and C5 formation [65].

**Figure 2:**
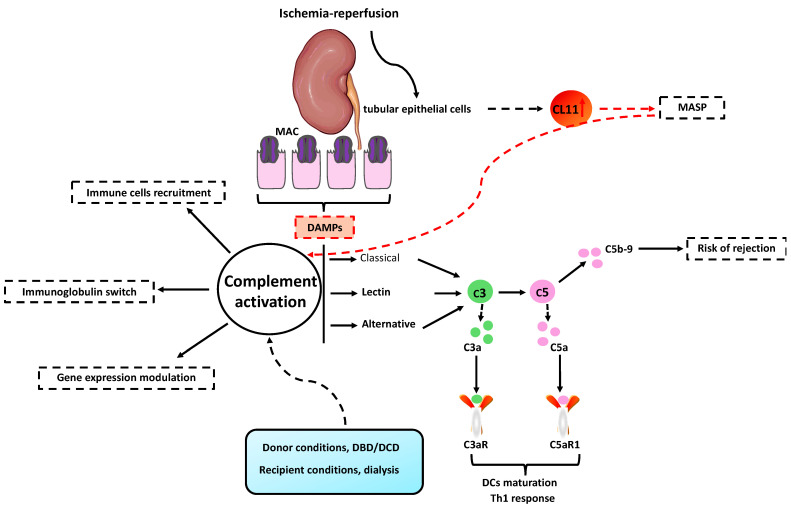
Complement system activation during IRI. IRI stimulates the complement system by releasing endogenous ligands (DAMPs) from acutely wounded tissue. The formation of the membrane attack complex (MAC) causes immediate damage to the kidney by inducing apoptosis in epithelial tubular cells. During IRI, tubular epithelial cells overexpress collectin-11 (CL11), which can activate mannan-binding serin protease (MASP). The main pathways involved in abnormal complement system activation during renal IRI are lectin and alternative. Higher serum levels of C5a and sC5b-9 were associated with a higher risk of rejection. C3aR/C5aR1 signaling is required for dendritic cells (DCs) maturation and the Th1 response. DAMPs—damage-associated molecular patterns; DBD—donation after brain death; DCD—donor after circulatory death.

Anaphylatoxins C3a and C5a are generated during the cascade and bind to G-protein coupled receptors (C3aR, C5aR1, and C5aR2) on a wide range of cells to increase vascular permeability, smooth muscle contraction, immune cell chemotaxis, and activation. APC priming activity on T cells is potentiated by increasing the expression of costimulatory molecules and antigen presentation. Another study highlighted that C3aR/C5aR1 signaling is essential for DCs maturation and for Th1 response. So, C5aR inhibition in both donors and recipients prolonged graft survival [66]. The IgG to IgM switch is also controlled by the complement receptor 2 (CCR2) in B cells [67]. Pericyte-to-myofibroblast (PMT) and endothelial-to-mesenchymal transition (EndMT) are crucial events during renal graft fibrosis upon IRI and could be orchestrated by the complement system [68]. Indeed, microvascular density and capillary lumen were preserved with C1 inhibitor (C1-INH) by limiting PMT [69]. Moreover, endothelial cells underwent downregulation of endothelial markers in favor of fibroblast ones as a result of the Akt pathway under C3a and C5a control. In vitro, epigenetic rearrangements (DNA methylation) have been described in tubular epithelial cells after C5aR activation during IRI. Chromatin remodeling has been associated with the downregulation of BCL9, CYP1B1, CDK6, and Klotho. Cell-cycle arrest markers (p21 and p53) are overexpressed. Klotho has anti-senescence and anti-fibrotic effects; therefore, its downregulation during IRI might contribute to early graft failure and chronic graft dysfunction [70,71]. The lack of C3aR and C5aR conferred protection against IRI, especially in case of C3aR/C5aR1 or C5aR1 deficiency. In mouse models of warm IRI, tissue inflammation and renal function impairment were reduced by inhibiting C5aR1 signaling using either a receptor-specific antagonist or a siRNA silencing strategy [72] (Figure 2).

## 6. Prevention and Therapeutic Perspective of IRI

Considering B cells’ role, their blockade might be effective in reducing IRI despite carrying a significant risk of immunocompromise and infections. So far, many drugs have been developed to target B cells: anti-CD20, anti-CD19, anti-BAFF (B cell activating factor), and Bruton’s tyrosine kinase (BTK) inhibitors (involved in BCR signaling). Non-selective removal of circulating macromolecules (such as antibodies, complement factors, coagulation factors, etc.) is referred to as plasma replacement. It is considered for antibody-mediated rejections (AMR), but its role in the prevention of IRI is limited [50].

Eculizumab (humanized anti-C5 monoclonal antibody) avoids the production of C5a and the deposition of MAC while keeping early complement activities. Numerous clinical studies have examined the role that complement inhibition (C1, C3, and C5) plays in preventing DGF and acute rejection following kidney transplantation, particularly AMR. Previous trials (NCT01403389, NCT01403389, and NCT01919346) already indicated that while peritransplant Eculizumab treatment is safe for transplant recipients of deceased donor kidneys, it is ineffective in preventing the formation of DGF [73]. The safety and efficacy of C1 esterase inhibitors (C1INH) have been investigated: treatment was associated with fewer dialysis sessions after transplantation, but the primary outcome (DGF prevention) was not met [74]. A better renal function associated with reduced apoptosis has been demonstrated by interfering gene expression (C3, Fas, caspase 3 and 8, Rel B, and CD40) with small interfering RNA (siRNA) and short hairpin RNA (shRNA) [75].

Further investigation might elucidate the role of the WnT/β-catenin pathway during renal IRI, considering its conflicting effects during ischemia and reperfusion phases in other organs. Different studies revealed that the Wnt pathway is active during the reperfusion phase instead of being inhibited as in the heart, liver, and brain [76,77]. Therefore, targeting the Wnt pathway and its crosstalk might represent a putative therapeutic strategy in the future.

Organ preservation and reperfusion techniques have achieved good results. The most used preservation solutions (Collins, Celsior, Histidine–Tryptophan–Ketoglutarate, University of Wisconsin, Hypertonic Citrate–Adenine) are characterized by the addition of metabolic intermediates with the aim of reducing calcium overload, increasing tissue ATP concentration, reabsorbing sodium, and reducing cell damage. Extensive research confirmed that ex vivo machine perfusion can be used as a weapon to treat organs before their implantation. By creating nearly physiological conditions, they can avoid systemic recipients’ treatment and donor therapy to reduce kidney injury. Typically, perfusion procedures are categorized based on the temperature of the perfusate: hypothermic and (sub)normothermic machines. The organ is pumped with perfusion solutions, which contain plenty of components such as oxygen, nutrition supplements, heparin, vasodilators, and antibiotics. These machines present a chance to expand the pool of transplantable organs, enhance outcomes, and overcome the difficulties associated with ECD or DCD kidneys. However, criteria for their use are currently pending. Many experimental and clinical trials assessed pharmacological and biological therapies to be further administered to the graft kidney during reperfusion (gases, cell, and gene therapy) [78].

## 7. Conclusions

In this review, we have described the entire complex pathophysiological mechanism of ischemia/reperfusion injury, which plays a pivotal role in the outcome of the graft. The activation of the innate and adaptive immune response raises the immunogenicity of the kidney, thus increasing the risk of T-cell and antibody-mediated rejection. No single therapeutic approach can fully address the effects of IRI, but rather, a combination of them (during organ retrieval, preservation, and in the peritransplantation period) might be beneficial. In the future, the modulation of the complement cascade will represent an attractive strategy to reduce the risk of IRI. However, additional experimental and clinical data are required to better understand the pathogenic events taking place in this complex process. Finally, high throughput analysis and multi-omics approaches will be critical in advancing therapeutic strategies to mitigate the effects of IRI during organ transplantation.

## Data Availability

No new data were created or analyzed in this study. Data sharing is not applicable to this article.
